# The Occurrence of Two Species of Entomophthorales (Entomophthoromycota), Pathogens of *Sitobion avenae* and *Myzus persicae* (Hemiptera: Aphididae), in Tunisia

**DOI:** 10.1155/2013/838145

**Published:** 2013-06-15

**Authors:** Ibtissem Ben Fekih, Sonia Boukhris-Bouhachem, Jørgen Eilenberg, Mohamed Bechir Allagui, Annette Bruun Jensen

**Affiliations:** ^1^Plant Protection Laboratory of National Institute of Agricultural Research of Tunisia, Rue Hédi Karray, 2049 Ariana, Tunisia; ^2^National Institute of Agronomy of Tunisia, University of Carthage, 43, Avenue Charles Nicolle, 1082 Cité Mahrajène, Tunisia; ^3^Department of Plant and Environmental Sciences, University of Copenhagen, Thorvaldsensvej 40, 1871 Frederiksberg, Denmark

## Abstract

The natural occurrence of entomophthoralean fungi pathogenic towards aphids on cereal and potato crops was investigated in the years 2009, 2010, and 2011. Infected aphids were sampled in three bioclimatic zones in Tunisia (Beja, Cap bon, and Kairouan) and fungal species were determined based on morphological characters such as shape, size, and number of nuclei in the primary conidia. Polymerase Chain Reaction (PCR) on the internal transcribed spacer 1 region (ITS1) was used to verify morphological determination. Both methods gave consistent results and we documented for the first time the natural occurrence of two fungal species from the order Entomophthorales (phylum Entomophthoromycota), *Pandora neoaphidis* and *Entomophthora planchoniana*. Both fungi were recorded on the aphid species *Sitobion avenae* and *Myzus persicae* on barley ears and potato leaves, respectively. Moreover, natural mixed infections by both species (*P. neoaphidis* and *E. planchoniana*) were documented on the target aphids. This investigation provides basic information of entomopathogenic fungi infecting economically important aphids in Tunisia.

## 1. Introduction

Aphids (Hemiptera: Aphididae) are one of the most important groups of insect pests in agriculture. They weaken their host plants in diverse ways by causing direct damage as phloem feeders and also by indirect damage as plant virus vectors [1]. A total of approximately 4000 aphid species have been described, of which 157 species have been reported in Tunisia [2]. Most of the identified species in Tunisia are considered to be pests and cause significant yield losses to important crops such as cereals [3]. Furthermore, dissemination of the *Potato Virus Y* (PVY) by aphids is considered as big problem in Tunisian potato fields [4]. The development of insecticide resistance among aphids has stimulated an interest in developing alternative methods of control [5]. Integrated pest management (IPM) can be seen as a sustainable control strategy to manage pest insects like aphids [6]. Field observations have shown that aphid populations are commonly regulated by a range of natural enemies, such as predators, parasitoids, and also fungal pathogens [7]. In temperate regions, fungal species from the phylum Entomophthoromycota are important pathogens of aphids [8–10]. Their ability to cause epizootics among their host insects within a short time makes them potentially valuable for pest control and by that an element in future IPM systems [11–15].

A new taxonomical revision assigned entomophthoralean fungi under the phylum Entomophthoromycota with three new orders, Entomophthorales, Neozygitales, and Basidiobolales [9]. The most common species worldwide infecting aphids belong to the Entomophthorales, particularly to the families Entomophthoraceae and Ancylistaceae, and the Neozygitales represented by the family Neozygitaceae [9]. The aphid pathogenic species have been documented almost worldwide with most records from temperate climatic zones [8, 16–18]. Little is known, however, about the natural occurrence of these fungal pathogens in North Africa. A previous study in Egypt recorded twelve entomopathogenic fungal species of which seven belonged to Entomophthoromycota [19]. The approach using entomopathogenic fungi in biological control is a new field in Tunisia. So far, only few studies on two *Fusarium* (Ascomycota) species on the artichoke aphid species *Capitophorus elaeagni* have been performed in Tunisia [20, 21]. Thus, basic knowledge about occurrence, distribution, and prevalence over time of entomophthoralean fungi in Tunisia is completely lacking. In this study we wanted to explore the natural occurrence of entomophthoralean fungi in relation to *Sitobion avenae* [22], among important aphid species infesting barley ears and *Myzus persicae* [22], a common pest aphids infesting potato fields in Tunisia.

## 2. Materials and Methods

### 2.1. Sampling

The investigations of mycoses in aphid population (apterae and alate specimens) were done in three regions of Tunisia: in the north west, Beja: site of the Regional Field Crop Research Center (36°44′00′′ N, 9°11′00′′ E), a sub-humid area; in the north east, Cap bon: Site of Soliman (36°40′40′′ N, 10°28′20′′ E), situated in the semiarid area of the region; in the center, Kairouan: Site of Sidi Mahmoud (35°37′07′′  N, 9°55′34′′ E), a continental zone with arid cold winter. Barley and potato fields infested with aphids were used for random sampling of fungal infected aphids between March and June of the years 2009, 2010, and 2011. Aphid cadavers with symptoms of fungal infection were placed into ventilated plastic boxes and carried to the laboratory.

### 2.2. Morphological Characterization

Fungus identification was based on the shape, size, and nuclei numbers in the primary conidia [23, 24]. The number of aphids subject to morphological identification of fungal infection was 730 for *M. persicae* and 980 for *S. avenae*. 

#### 2.2.1. Sample Preparation

The aphid cadavers with fresh conidiophores were inverted over a glass slide in moist boxes at 20°C to allow conidia ejection for first 5 hours and then 8 hours. Subsequently, some of the cadavers were stored individually in 96% ethanol to be used in later molecular examination. Light and electron microscopic studies were used for the morphological determination of the fungal species.

#### 2.2.2. Light Microscopy

Conidia projected on the slide were mounted in either lactic acid or aceto-orcein for measuring size or counting nuclei numbers, respectively. The shape and size (length and width) and the nuclei number per conidia of twenty randomly chosen conidia per aphid were measured and counted on a computer screen coupled to an Olympus microscope at 400x magnification. 20 aphids per crop were used.

#### 2.2.3. Scanning Electron Microscopy

Infected aphids were examined with an environmental scanning electron microscope (ESEM) aiming to obtain information about detailed structures of the fungi. Samples were carbon coated using a conductive carbon disk and observation was done through the ESEM model QUANTA 200I-D7827 with tungsten (W) filament electron source.

### 2.3. Molecular Characterization: DNA Extraction and PCR Amplification

Genomic DNA from 14 infected aphids (*Myzus persicae* and *Sitobion avenae*) and one healthy *Sitobion avenae* taken from rearing chamber and considered as a negative control was extracted using a Chelex extraction protocol [25]. DNA extraction was done by adding 20 *µ*L phosphate buffered saline (PBS PH7.2) and 5 *µ*L proteinase K (10 mg/mL) to each 1.5 mL Eppendorf tube and the aphids were homogenized with a DNA-free pestle. After a quick spin at 10,500 ×g for 30 s, 100–200 *µ*L (depending on aphid size) of a 10% Chelex solution was added and the samples were incubated overnight at 56°C. Next day the samples were incubated at 94°C for 15 min and after a spin at 10,500 ×g for 30 s, the supernatants were transferred to new Eppendorf tubes and stored at −20°C. 

PCR was performed on the internal transcribed spacer 1 (ITS 1) using two genus specific forward primers for *Entomophthora* and *Pandora*, respectively: Ml2: 5′-GCAACGGATCATCATGTAA-3′ and PnCNf: 5′-TTTGGGTTTAAATAGAAGGTTGA-3′ and reverse primers Nu-5.8S-3′: 5′-ACTACGTTCTTCATCGATGA-3′ [10] and PnCNr: 5′-AGGCAAAGCCTAGAGCACTT-3′ (unpublished). The primers were chosen to detect and confirm the identity of the fungi infecting the field collected aphids.

Positive DNA controls provided from ARSEF Collection of Entomopathogenic Fungal Cultures: ARSEF 2583: *Pandora neoaphidis*, isolated from the aphid species *Acyrthosiphon pisum* (USA, 1988) and ARSEF 6918: *Entomophthora muscae*, isolated from the Dipteria *Coenosia tigrina* (Denmark, 1999) and negative water controls were included in each set of PCR reactions.

PCR amplifications were performed in 50 *µ*L reaction volumes containing 2 *µ*L of chelex-extracted DNA 1 : 1 or diluted 1 : 10, 10 *µ*L Phusion HF Buffer (5 × 7.5 mM MgCl_2_), 10 mM dNTPs, 0.5 *µ*M of each primer, 0.5 U Phusion High-Fidelity DNA Polymerase (Finnzymes, Espoo, FI). For both primers, the PCR conditions were denaturation at 98°C for 30 s, followed by 38 cycles of denaturation at 98°C for 10 s, annealing at 55°C for ML2 or 60°C for PnCNf for 20 s, and extension at 72°C for 1 min, with a final extension at 72°C for 10 min. The size of the PCR amplifications was estimated by electrophoresis on a 1.5% agarose gel in 0.5 × TBE, and the products visualized with EZ-Vision (AMRESCO LLC, USA).

Sequencing was used to verify the species identity based on the length of the PCR product, in particular for the *P. neoaphidis* primers PnCNf/PnCNr which were tested for the first time in this study. Prior to sequencing the PCR products were purified with Qiagen kit. The different PCR products were sequenced by Eurofins MWG and a sequences similarity search using NCBI BLAST was performed in Genbank.

## 3. Results and Discussion

Our study documented for the first time in Tunisia fungal species within the phylum Entomophthoromycota, family Entomophthoraceae [9]. We identified two species of the subfamilies Erynioideae and Entomophthoroideae, respectively: *Pandora neoaphidis*, (Remaudière and Hennebert) [26] and *Entomophthora planchoniana* [27] found on *M. persicae *and *S. avenae,* respectively.

### 3.1. Morphological Characterization

**Figure 1:**
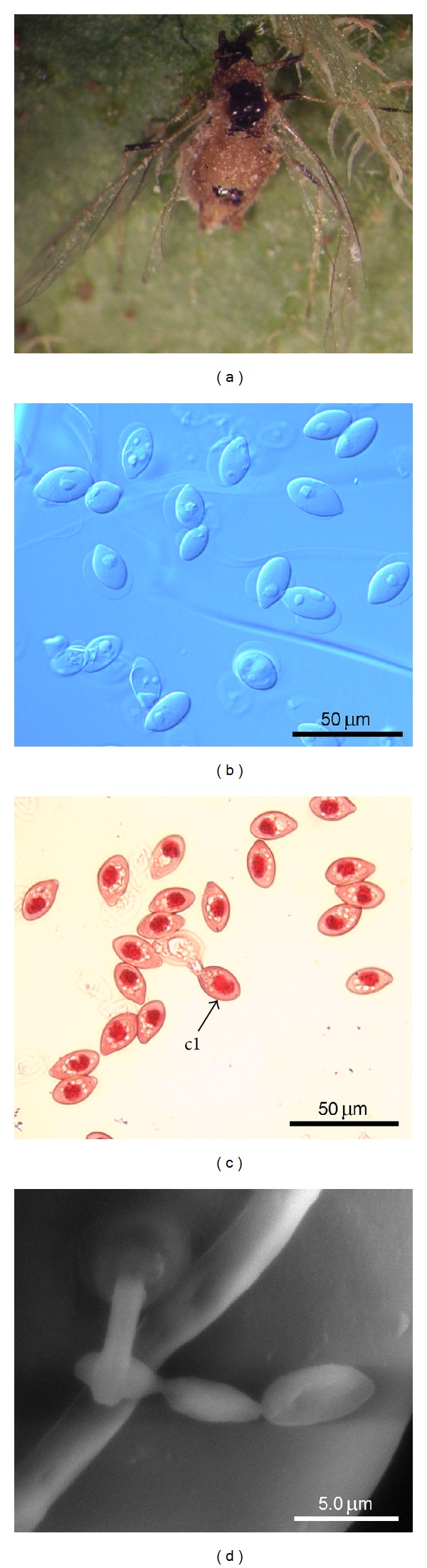
*Pandora neoaphidis.* (a) *Myzus persicae* killed and with fungus outgrowth, (b) primary conidia (stained by lactic acid), (c) primary conidia (stained by aceto-orceine) documenting one nucleus per conidium. (c1) Secondary conidium being produced by primary conidium. (d) SEM image of a germinating primary conidium on host cuticle.

The species *P. neoaphidis* is characterized by visible ellipsoid mononucleate primary conidia [23, 24] (Figure 1). The *Pandora* conidia from the Tunisian material contained one nucleus each and measured in length and width 22.1–30.9 × 15.6–18.8, respectively (Table 1). This is well within the known range of *P. neoaphidis* conidia; however, the conidia obtained during this investigation tended to be a bit larger when compared to data in [23].

**Figure 2:**
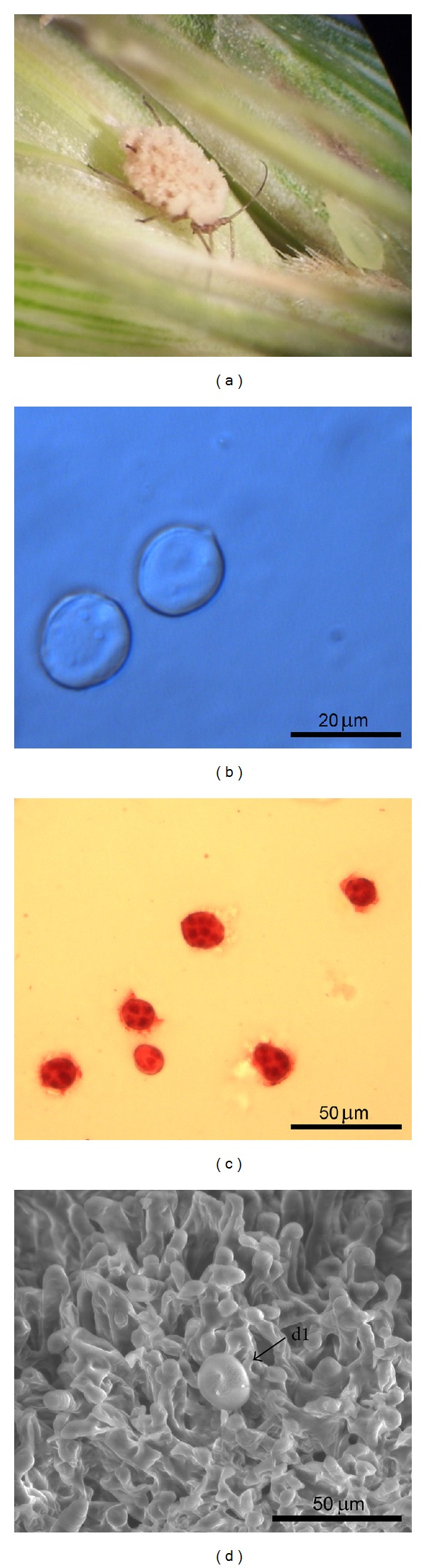
*Entomophthora planchoniana*. (a) *Sitobion avenae* killed and with fungus outgrowth. (b) Primary conidia (stained by lactic acid). (c) Primary conidia (stained by aceto-orceine). (d) SEM image of hyphae, conidiophores, and one visible conidium (d1).

The species *E. planchoniana* is characterized by bell shaped plurinucleate primary conidia with sharp apical point and a broad flattened papilla [24] (Figure 2). The *Entomophthora* conidia from the Tunisian material contained between 4 and 7 nuclei and measured in length and width 16.5–29.9 × 11.7–18.2, respectively (Table 1). Nuclei numbers and conidia dimensions of this species are thus within the description of *E. planchoniana* [24].

**Figure 3:**
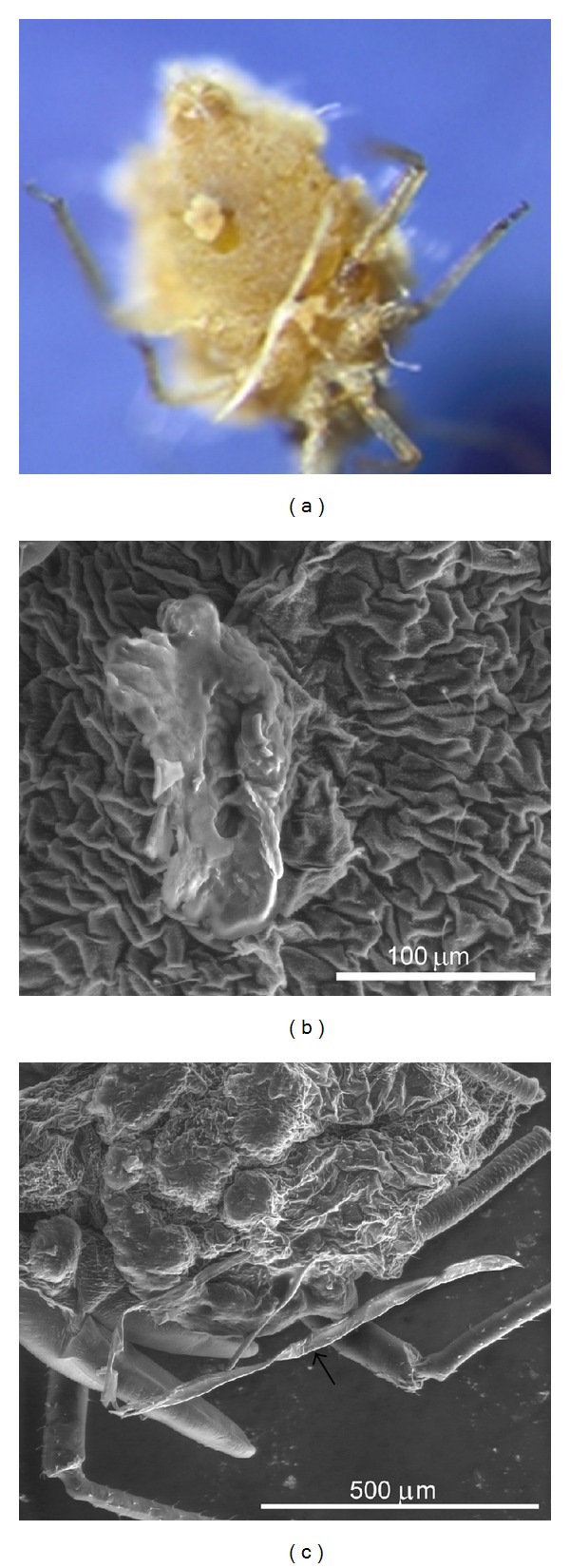
Monohyphal rhizoids. (a) *Entomophthora planchoniana* rhizoids with disc-like ending emerging from the ventral abdominal region of infected *Rhopalosiphum padi*. (b) SEM Image of *E. planchoniana* rhizoids. (c) *Pandora neoaphidis* rhizoids with irregularly terminal branches.

In this study, primary conidia were the main fungal structure used for species identification. However, another fungal structure emerging from the host aphid cadavers named rhizoids ensuring the attachment of the aphids to the plants was also documented in this study. The presence or absence of rhizoids and their characters (monohyphal or compound) with or without specialized holdfasts should be taken into consideration during the fungus identification [15]. *E. planchoniana* have particular monohyphal rhizoids with disc-like ending which is a characteristic of the species (Figures 3(a) and 3(b)). However, *P. neoaphidis* rhizoids are monohyphal ending with irregularly terminal branches (Figure 3(c)).

### 3.2. Molecular Analysis

**Figure 4:**
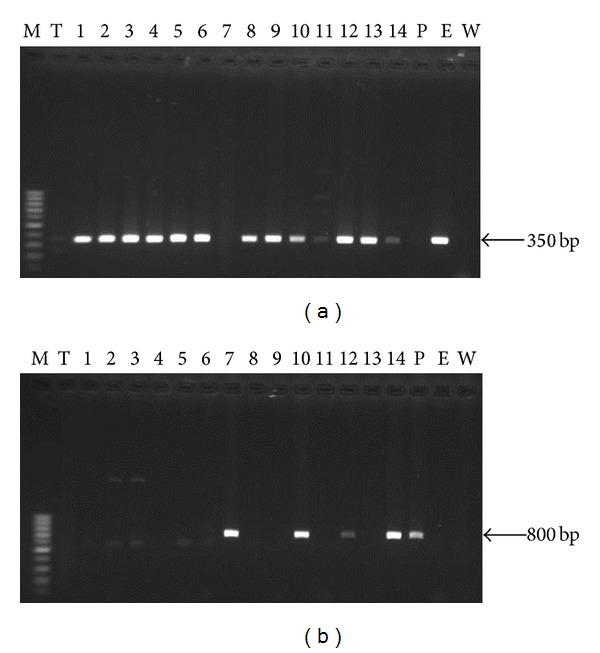
Amplification of ITS1 region using (a) the *Entomophthora* specific primer set ML2/Nu-5.8S-3′ and (b) *Pandora neoaphidis* primers PnCNf / PnCNr, using DNA extracted from a healthy aphid (T), 14 entomophthoralean killed cadavers (1–14), *Pandora neoaphidis* DNA (P), and *Entomophthora muscae* DNA (E). Size marker (MW) = 100 pb.

We showed that two genus specific ITS 1 primer sets ML2/Nu-5.8S-3′ (*Entomophthora*) and PnCNf/PnCNr (*Pandora*) worked well on the aphid cadavers collected in Tunisia. The amplification of the ITS1 region from 14 infected aphids using the two primers sets showed different profiles. The *Entomophthora* primers amplified a single amplicons with expected size estimated to 350 bp for 13 samples which was the same size for the positive control ARSEF 6918 (E) (Figure 4(a)). Sample numbers 11 and 14 show less intense amplicons which could be related to the depletion of fungal material in the hosts due to the full discharge of *Entomophthora* conidia. This was congruent with the morphological analysis of conidia from the same infected aphid cadavers that were all identified as *E. planchoniana *(Table 2). DNA extractions from the same 14 aphids were also screened with the *Pandora* primers. One clear single band was produced from 4 infected cadavers with same size for the positive control ARSEF 2583 (P). The amplification profile gave strong amplicons for the samples number 7, 10, and 14 whereas it was less intense for the aphid sample number 12 (Figure 4(b)). This was congruent with the morphological analysis of conidia from the same infected aphid cadavers that were all identified as *P. neoaphidis *(Table 2). No amplification product was obtained from uninfected aphid and the water (W) both considered as negative control.

Similarity search of nucleotide sequences in GenBank shows that all *E. planchoniana* sequences were 100 percent similar to *E. planchoniana* isolate. For *P. neoaphidis* all the sequence were 99 percent similar to many *P. neoaphidis* isolates in Genbank. This is in favor of future use of PnCNf/PnCNr for molecular characterization of *P. neoaphidis* isolates and probably also other *Pandora* species.

PCR profiles indicated possible mixed fungal infections in samples 10, 12, and 14 (Figures 4(a) and 4(b)). The morphological examination of the conidia projected from those aphid samples showed the presence of both *P. neoaphidis* and *E. planchoniana*. This “concomitant” or “mixed infection” seems to be relatively common in nature in our pest-pathogen systems and the frequency of such mixed infections may actually be influenced by both environmental conditions and other factors [28, 29].

The simultaneous usage of both morphological and molecular methods gave a very strong background both with respect to the correct identification of *P. neoaphidis* and *E. planchoniana* and with respect to future studies determining the full spectrum of entomophthoralean fungi infecting aphids and their wider distribution of these fungi in Tunisia.

Both species were found in all the bioclimatic zones included in this study: an arid region (Kairouan), a sub-humid region (Beja), and a semi-arid region (Soliman, Cap bon). Cereal and potato areas are mainly situated in the north and center of Tunisia, where the climate switch from subhumid to arid which is considered suitable environment for the development of entomophthoralean infection. The occurrence of *P. neoaphidis* and *E. planchoniana* in the investigated regions seems to be in concordance with their distribution in different bioclimatic zones. They have both a worldwide distribution (Europe, Australia, North and South America, North and South Africa, and Asia), and while *P. neoaphidis *is common when the temperature is moderate and the humidity high, *E. planchoniana* can be common in dry and moderately humid environment [8, 16].

Aphid pathogenic fungal species belong either to the phylum Ascomycota within the order Hypocreales (genera* Beauveria, Fusarium, Paecilomyces, Lecanicillium,* and others) or to the phylum Entomophthoromycota [9, 23]. Previous investigation of pathogens on the artichoke aphid species *Capitophorus elaeagni* in Tunisia reported two Fusarium species (*F. sacchari and F. semitectum*), both showing potential against different aphid species [20, 21]. The investigation was done in two region of Tunisia: Bizerte (humid zone) and Sousse (semiarid zone). Interestingly, no entomophthoralean species was recorded by these authors, despite their widespread occurrence in such bioclimatic zones documented in our studies. Which might be due to the sampling strategy.

Species from the order Entomophthorales are host-specific with high potential to regulate aphid populations in field crops. Both identified species are considered to be dominant among 30 species of Entomophthorales infecting a wide range of hosts from Aphidoidea (Hemiptera) [8, 16]. However, obstacles related to the mass production and inoculums formulation of both fungi are still not solved [8]. To date, biological control guidelines of aphids with Entomophthorales highlight often the conservation biological control approach which enhances the natural occurring of those pathogens [14].

## 4. Conclusion

The present research provides fundamental information on the occurrence of fungal entomopathogens infecting economically important aphids in Tunisia. Further surveys will be considered to explore other pathogen species and prevalence studies will be adopted to explore the potential of this order. Such information could be used in the establishment of a framework for a national program of integrated pest management. In addition, it will improve our understanding of the worldwide distribution of aphid pathogenic Entomophthoromycota in particular from North Africa.

## Figures and Tables

**Table 1:** Number of nuclei and dimensions (in *µ*m) of primary conidia of *Pandora neoaphidis* and *Entomophthora* *planchoniana. *

Fungal species	Number of nuclei	Length × width of the primary conidia	Reference
*P. neoaphidis *	1	15–40 × 9–16	Humber [23]
	1	22.1–30.9 × 15.6–18.8	This study

*E. planchoniana *	4–8	15–20 × 12–16	Keller [24]
	4–7	16.5–29.9 × 11.7–18.2	This study

**Table 2:** Fungal identification using morphological and molecular analysis.

Aphids code	Aphid species	Host plant	Sampling areas	Morphological analysis		Molecular analysis	
				*P. * *neoaphidis *	*E. * *planchoniana *	*P. * *neoaphidis *	*E. * *planchoniana *
1	*S. avenae *	Barely	Beja	−	+	−	+
2	*S. avenae *	Barely	Beja	−	+	−	+
3	*S. avenae *	Barely	Beja	−	+	−	+
4	*S. avenae *	Barely	Soliman	−	+	−	+
5	*M. persicae *	Potato	Soliman	−	+	−	+
6	*M. persicae *	Potato	Kairouan	−	+	−	+
7	*S. avenae *	Barely	Beja	+	−	+	−
8	*S. avenae *	Barely	Soliman	−	+	−	+
9	*M. persicae *	Potato	Kairouan	−	+	−	+
10	*M. p* *er* *si* *ca* e*	Potato	Soliman	+	+	+	+
11	*M. persicae *	Potato	Soliman	−	+	−	+
12	*M. p* *er* *si* *ca* e*	Potato	Soliman	+	+	+	+
13	*M. persicae *	Potato	Kairouan	−	+	−	+
14	*S. a* *ve* *na* e*	Barely	Soliman	+	+	+	+

(+) Documented fungi species; (−) undocumented fungi species.

*Aphids with mixed infection.
